# Insulin Promotes Glucose Consumption via Regulation of miR-99a/mTOR/PKM2 Pathway

**DOI:** 10.1371/journal.pone.0064924

**Published:** 2013-06-10

**Authors:** Wei Li, Jing Wang, Qiu-Dan Chen, Xu Qian, Qi Li, Yu Yin, Zhu-Mei Shi, Lin Wang, Jie Lin, Ling-Zhi Liu, Bing-Hua Jiang

**Affiliations:** 1 Department of Pathology, Cancer Center, Nanjing Medical University, Nanjing, China; 2 Department of Neurosurgery, The First Affiliated Hospital of Nanjing Medical University, Nanjing, China; 3 Department of Pathology, Anatomy and Cell Biology, Kimmel Cancer Center, Thomas Jefferson University, Philadelphia, Pennsylvania, United States of America; 4 Faculty of Software, Fujian Normal University, Fuzhou, China; Virgen Macarena University Hospital, School of Medicine, Spain

## Abstract

Insulin is known to regulate multiple cellular functions and is used for the treatment of diabetes. MicroRNAs have been demonstrated to be involved in many human diseases, including Type 2 diabetes. In this study, we showed that insulin decreased miR-99a expression levels, but induced glucose consumption and lactate production, and increased the expression of mTOR, HIF-1α and PKM2 in HepG2 and HL7702 cells. Forced expression of miR-99a or rapamycin treatment blocked insulin-induced PKM2 and HIF-1α expression, and glucose consumption and lactate production. Meanwhile, knockdown of HIF-1α inhibited PKM2 expression and insulin-induced glucose consumption. Taken together, these findings will reveal the role and mechanism ofinsulin in regulating glycolytic activities via miR-99a/mTOR.

## Introduction

Insulin, secreted from pancreatic B-cells, is an important peptide hormone and usually used for the treatment of diabetes [1], [2]. Insulin is known to regulate multiple cellular functions such as synthesis of sugar, fat and protein, and is central in regulating carbohydrate and fat metabolism [3], [4]. MicroRNAs (miRNAs) are small non-coding RNAs with 19–22 bp length and post-transcriptionally regulate gene expression by binding to 3′-untranslated regions (3′-UTRs) of target mRNAs [5], [6]. MiRNAs have been demonstrated to be involved in many human diseases [5], [6], [7], including type 2 diabetes [8], [9], [10]. Many studies showed that miRNAs can regulate insulin secretion. For example, miR-33a decreased glucose-stimulated insulin secretion via reducing expression of ABCA1(ATP-binding cassette transporter A1) [11]. Over-expression of miR-375 reduced glucose-induced insulin secretion by regulating myotrophin (Mtpn) [12]. Recent studies found that the expression of miRNAs can be regulated by insulin such as miR-208 which played a role in insulin-induced VSMC proliferation by p21, a key member of CDK-inhibitory protein family [13]. However, little is known about the roles of miRNAs in insulin-regulated cellular functions.

Mammalian target of rapamycin (mTOR) is a serine/threonine kinase which plays critical roles in regulating protein synthesis, ribosomal protein translation, cap-dependent translation and mTOR signaling is associated with cell growth, proliferation, apoptosis [14], [15], [16]. Emerging evidence demonstrates that mTOR are regulated by miRNAs. Through mTOR pathway, miR-204 regulated cancer cell migration and invasion [17], and miR-100 induced cell-cycle arrest to suppress cell proliferation and motility in bladder cancer cells [18]. These results are consistent with our observation that mTOR was inhibited by miR-99a in hepatocellular carcinoma cells (HCC). It has been demonstrated that insulin can regulate glucose metabolism by activating mTOR pathway [19], [20]. The liver is known to be important in regulating glucose metabolism in response to growth factors and insulin treatment [21], [22]. With the co-treatment of saffron and insulin, mTOR pathway improves the insulin sensitivity [23]. mTOR pathway is required to sustain glucose metabolism and glycolysis, and is important in the transcriptional program of glucose transporters and multiple rate-limiting glycolytic enzymes [24]. Pyruvate kinase M2 (PKM2), which is expressed in fetal tissues, plays a critical role in glycolytic pathway as a rate-limiting enzyme, and catalyzes the dephosphorylation of phosphoenolpyruvate topyruvate [25], [26]. Expression levels of PKM2 are upregulated in human cancer cells [27], [28], PKM2 promotes glucose metabolism in cancer cells by stimulating transactivation of glycolytic genes [25], [29]. However, itremains to be defined how PKM2 is regulated in cells. PKM2 emerges as an important regulator in glucose metabolism during cancer development and tumor growth [30], [31], [32]. Recent studies identified that PKM2 was regulated by AKT/mTORsignaling pathway in cell growth, survival, and metabolism [30], [33]. It was demonstrated that mTOR pathway upregulated glycolysis in hepatocellular carcinoma [34]. However, there is little information whether miRNA(s) is/are involved in insulin-regulated mTOR/PKM2 pathway. We tested the effect of insulin on the known and unknown miRNAs that target mTOR and found out that miR-99a were regulated by insulin. In this study, we plan to determine 1) whether insulin regulates mTOR through miR-99a; 2) whether insulin regulates glycolytic activities via miR-99a/mTOR; 3) what is the downstream effectors of mTOR for mediating insulin-induced glycolytic activities.

## Materials and Methods

### 2.1 Reagents and Cell Lines

Antibodies against mTOR and PKM2 were purchased from Cell Signaling Technology (Beverly, MA, USA). Antibody against HIF-1α was purchased from Bioworld technology (Louis Park, MN, USA) and BD Biosciences (Franklin Lakes, NJ, USA). Antibodies against β-actin and GAPDH were purchased from Sigma-Aldrich Inc. (St. Louis, MO, USA) and Kangchen Bio-tech Inc. (Shanghai, China), respectively. Rapamycin was obtained from Sigma-Aldrich and dissolved in DMSO. Insulin was obtained from Biosharp (Korea) and dissolved in 50% Glycerol. Lipofectamine and TRIzol reagent were provided by Life Technologies(Grand Island, NY, USA). MiR-99a mimics was provided by GenePharma (Shanghai, China). HEK293, HepG2 cells were obtained from American Type Culture Collection, HL7702 cellswere obtained from Cell bank of Chinese Academy of Science (Shanghai, China). Cells were cultured according to the manufacturer’s instructions.

### 2.2 Cell Culture and Treatment

Human liver cells HL7702 and human liver carcinoma cell HepG2 were cultured in cell medium and incubated in a humidified incubator containing 5% CO_2_ at 37°C. HL7702 cells were cultured in RPMI 1640 medium with 10% fetal bovine serum (FBS), 100 units/ml penicillin, 100 µg/ml streptomycin, whereasHepG2 cells were cultured in DMEM medium with 10% FBS, 100 units/ml penicillin and 100 µg/ml streptomycin. For insulin treatment, cells were seeded in 6-well plates or 24-well plates and starved in low-glucose, serum-free medium for 18 hours followed by treatment with insulin (200 nM) or basal medium for 6 hours. Before treated as above, some cells were transfected with microRNA mimics (40 nM), siHIF-1α (40 nM) or negative control oligomer for 24 hours or pretreated with rapamycin (5 nM) for 30 minutes.

### 2.3 Cell Transfection

Hsa-miR-99a or hsa-miR-SCR mimics were transfected into HepG2 and HL7702 cells using lipofectamine reagent. Transfection complexes were prepared according to the manufacturer’s instructions. The final concentrations of hsa-miR-99a or hsa-miR-SCR mimics for the transfection were 40 nM. Similar method was used to transfect siHIF-1α (40 nM) or siSCR into HepG2 cells as above.

### 2.4 RNA Isolation and Quantitative Real-time PCR

Total RNAs were extracted from cells using TRIzol reagent. Stem-loop reverse transcriptase (RT) was carried out using Primescript RT Regent Kit (Takara, Dalian, China) according to the manufacturer’s instruction. The sequences of the RT primers were as following: miR-99a-RT, 5′-CGTTGGTTGTCCCATAGACTCACAAGATC-3′; U6-RT, 5′-TGGTGTCGTGGAGTCG-3′. Real-time PCR was performed using SYBR Premix DimerEraser (Takara, Dalian, China) on a 7900 HT system, and RNA input was normalized to the level of human U6 snRNA. The qPCR primers were as following: miR-99a primers: sense: 5′-GGCAAACCCGTAGATCCGA-3′; anti-sense: 5′-TCCGTTGGTTGTCCCATAGACT-3′. U6 snRNA primers: Sense: 5′-CTCGCTTCGGCAGCACA-3′; anti-sense: 5′-AACGCTTCACGAATTTGCGT-3′. The relative expressions of miR-99a were normalized to the levels of U6 and analyzed via the comparative cycle threshold method (2^−ΔΔCT^).

### 2.5 Protein Extraction and Immunoblotting Analysis

Cells with specific treatments were lysed in radioimmunoprecipitation assay (RIPA) buffer (100 mMTris, pH 7.4, 150 mMNaCl, 5 mM EDTA, 1% Triton X-100, 1% deoxycholate acid, 0.1% SDS) supplemented with protease inhibitors (2 mMphenylmethylsulfonyl fluoride, 1 mM sodium orthovanadate, 2 mM DTT, 2 mMleupeptin, 2 mMpepstatin). The homogenates were centrifugated at 12,000 rpm for 15 min at 4°C, and the supernatants were collected and stored at −80°C. Protein concentrations were measured by BCA assay. SDS-polyacrylamide gel electrophoresis (SDS-PAGE) was used to separate protein extracts and then the gel was transferred to nitrocellulose membranes. Membranes were blocked with 5% nonfat dry milk in 1× PBS containing 0.05% Tween-20 for 2 hours. The protein bands were incubated with antibodies against mTOR, PKM2,HIF-1α, GAPDH or β-actin and then secondary antibody conjugated to horseradish peroxidase. The reaction signal was detected with ECL Detection System (Thermo Scientific, MA, USA).

### 2.6 Metabolism Assays

HepG2 and HL7702 cells were seeded into 24-well plate with 200 µl media each well. To determine the levels of glucose and lactate in HepG2 and HL7702 cells, the supernatants of cell culture media were collected and assayed for glucose and lactate levels by using glucose assay kit and lactate assay kit (BioVision, San Francisco, USA) according to the manufacturer’s instructions. The values at different time periods were analyzed by the OD values. Glucose consumption and lactate production were calculated based on the standard curve, and normalized to the cell number.

### 2.7 Vector Construction and Luciferase Assay

The mTOR 3′-UTR sequence was amplified from human cDNAs by PCR using the following primers: mTOR forward primer, 5′-GCGAGCTCCTTTAGAAATACGGGTTTTGACTTA-3′; mTOR reverse primer, 5′-GCAAGCTTGCCGAGGCTGCCAGCGATCTGAATA-3′. For the mTOR mutagenesis, the sequences complementary to the binding site of miR-99a in the 3′-UTR (UACGGGU) was replaced by AUGCCCA, respectively [35]. The wildtype and mutated 3′-UTR regions of mTOR were cloned into pMIR-REPORT miRNA reporter vector using the Sacl and HindIII sites. These constructs were validated by DNA sequencing.

HEK-293 cells were seeded into a 24-well plate. After cultured for 24 hours, cells were co-transfected with wild-type or mutant mTOR 3′-UTR reporter plasmid and pRL-TK plasmid (as control), or transfected with pre-miR-99a and miR-scrambled control precursors (miR-SCR). Luciferase assays were performed 48 hours after tranfection using the Dual Luciferase Reporter Assay System (Promega, WI, USA).

### 2.8 Statistical Analysis

In this study, each value was obtained from at least three independent experiments and presented as means ± SE. Student’s unpaired *t* test. The data were analyzed by ANOVA for groups with 3 and more treatments, and by Student’s unpaired *t* test for groups with two treatments The values were considered significantly different between groups when *P*<0.05.

## Results

### Insulin Inhibited miR-99a Expression, but Induced Glucose Consumption and Lactate Production, and Increased the Expression Levels of mTOR, Phosphorylated mTOR (p-mTOR), HIF-1α and PKM2 in HepG2 and HL7702 Cells

**Figure 1:**
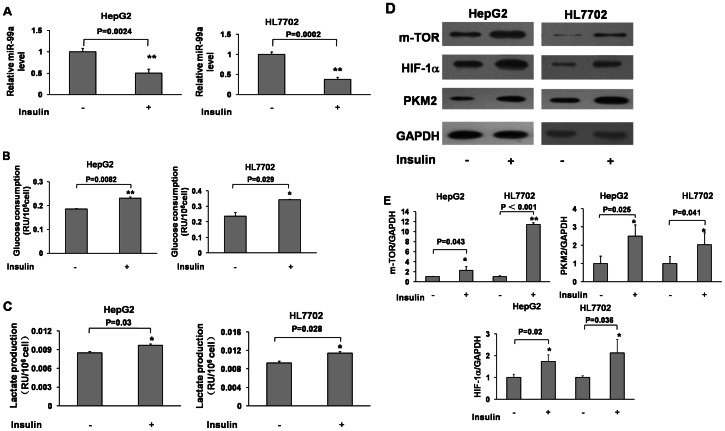
Insulin inhibited miR-99a expression, but induced glucose consumption and lactate production, and increased the expression of mTOR, HIF-1α and PKM2 in HepG2 and HL7702 cells. HepG2 and HL7702 cells were starved in serum-free medium for 18 h, and then treated with insulin (200 nM) for 6 h. (A) Cells were collected and subjected to miR-99a and U6 expression by qRT-PCR. Relative miR-99a expression levels were normalized to U6. Data were presented by mean±SE (n = 3). **, indicates significant decrease compared to the cells without insulin treatment (p<0.01). (B) The supernatant of cultured medium was collected and subjected to glucose assay of cells treated by insulin. Data were presented by mean±SE (n = 3). * and **, indicate significant increase compared to the cells without insulin treatment (p<0.05 and p<0.01). (C) The supernatant of cultured medium was collected and subjected to lactate assay. Data were presented by mean±SE (n = 3). *, indicates significant increase compared to the cells without insulin treatment (p<0.05). (D) Cell pellets were collected and subjected to immunoblotting analysis of mTOR, HIF-1α, PKM2 and GAPDH. (E) The density of protein levels of above was quantified by ImageJ software and normalized to the level of GAPDH. Data were presented by mean±SE (n = 3). * and **, indicate significant increase compared to the cells without insulin treatment (p<0.05 and p<0.01).

Recent studies have shown that mTOR is involved in glucose metabolism [36], [37], [38], [39]. Lactate, a product from glucose metabolism, is produced from pyruvate conversion. In order to test whether certain miRNA(s) could affect insulin-mediated glucose metabolism, we tested the potential miRNAs that target mTOR and interestingly found that miR-99a was downregulated by 2-fold in both HepG2 and HL7702 cells after insulin treatment (Fig. 1A). Meanwhile, insulin induced glucose consumption and lactate production to more than 1.5-fold in both cells (Figs. 1B and 1C). mTORis an important regulator in insulin-induced activation of S6 and 4EBP1 signals [40]. Here we showed that insulin treatment increased mTOR expression at total and phosphorylated protein levels (Figs. 1D, 1E, and S1). Consistent with previous studies showing that HIF-1 is one of the downstream molecules regulated by mTOR/p70S6K1, insulin treatment stimulated HIF-1α expression. PKM2, a critical enzyme for aerobic glycolysis and tumor growth [14], [30], was also up-regulated by insulin treatment (Figs. 1D and 1E). These results suggest that insulin may down-regulate miR-99a, up-regulate mTOR, HIF-1α, PKM2 expression and promoteglycolytic activities.

### Insulin Induced mTOR Expression Levels, Glucose Consumption and Lactate Production via miR-99a

**Figure 2:**
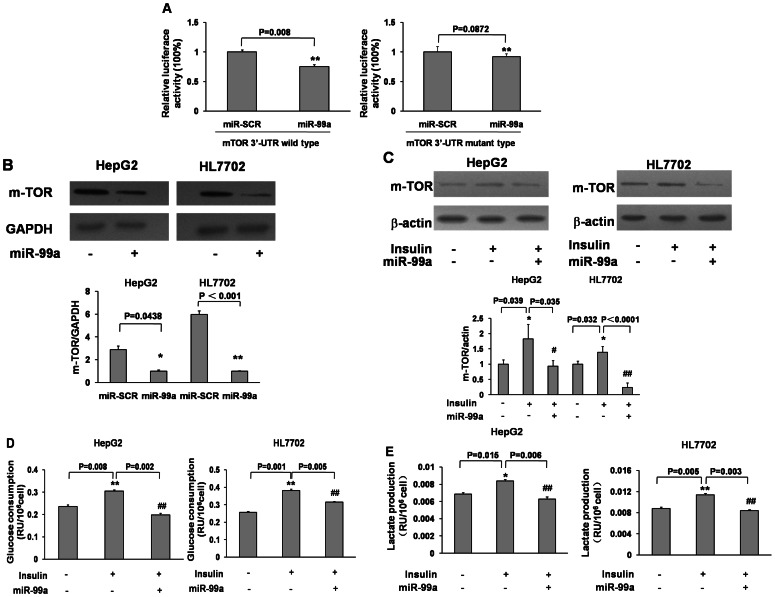
Insulin induced mTOR expression, glucose consumption and lactate production via miR-99a. (A) The luciferase reporters containing the wild type and mutant 3′-UTR of mTOR at predicted miR-99a binding site were constructed and verified by sequencing. HEK-293 cells were transfected with wild type or mutant reporter plasmid, scrambled control miRNA (miR-SCR) or miR-99a precursor, and pRL-TK plasmid, and the relative luciferase activity was calculated as the ratio of firefly/renilla activities and normalized to that of the control. Data were presented by mean±SE (n = 3). **, indicates significant decrease compared to the control (p<0.01). (B) Cells were transfected with miR-99a or miR-SCR at 40 nM. After 72 h, the expression of mTOR and GAPDH was determined by immunoblotting (up panel). Relative quantitative levels of mTOR were measured as above (bottom panel). * and **, indicate significant decrease compared to the miR-SCR group (p<0.05 and p<0.01). (C) HepG2 and HL7702 cells were transfected as above, then starved and treated with or without insulin. Cell pellets were collected and the relative density of mTOR expression was determined. The data were analyzed by ANOVA. *, indicates significant increase compared to the control without insulin treatment (p<0.05); ^#^ and ^##^, indicate significant decrease compared to miR-SCR and insulin treatment (p<0.05 and p<0.01). The glucose consumption (D) and lactate production (E) were tested in supernatant of treated cells as above. * and **, indicate significant increase compared to the control without insulin treatment (p<0.05 and p<0.01); ^##^, indicates significant decrease compared to miR-SCR and insulin treatment (p<0.01).

We found a negative correlation between miR-99a and mTOR expression levels under the insulin treatment. We hypothesize that insulin regulates glucose consumption through mTOR/PKM2 pathway *via* miR-99a. To determine whether mTOR is a direct target of miR-99a, the 3′-UTR region of mTOR was cloned into pMIR-REPORT miRNA reporter vector. Consistent with a recent study showing that miR-99a directly targets and regulates mTOR in childhood adrenocortical tumors [41], we found that forced expression of miR-99a suppressed luciferase activity of the wild type, but not the mutant mTOR reporter (Fig. 2A) and mTOR expression at protein level (Fig. 2B). To further study whether insulin stimulates glucose consumption via regulating miR-99a and mTOR, HepG2 and HL7702 cells were transfected with pre-miR-99a and negative control of miRNA precursor (miR-SCR). As we expected, miR-99a overexpression inhibited insulin-induced total mTOR and p-mTOR expression levels (Figs. 2C and S1). In agreement with the mTOR suppression, miR-99a overexpression also inhibited insulin-induced glucose consumption (Fig. 2D) and lactate production by 30–40% and 30–35%, respectively in HepG2 and HL7702 cells (Fig. 2E). These results indicate that miR-99a is an important regulator in insulin-regulated glycolytic activities through directly targeting mTOR.

### Overexpression of miR-99a or Rapamycin Treatment Inhibited Insulin-induced PKM2 and HIF-1α Expression, and Glucose Consumption and Lactate Production

**Figure 3:**
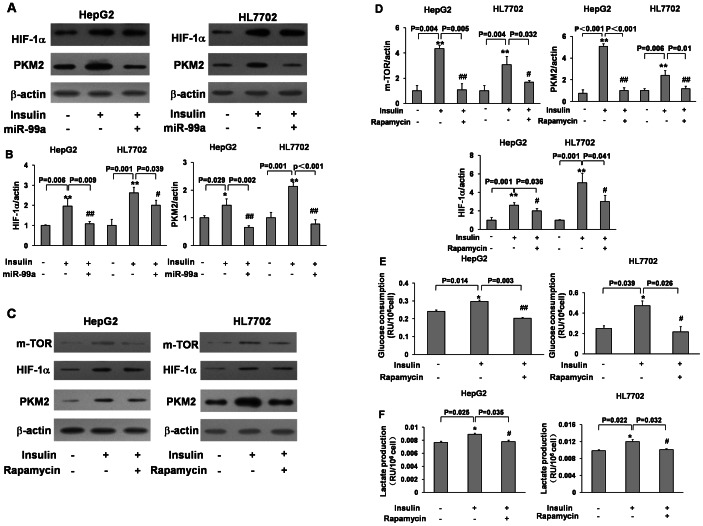
Overexpression of miR-99a or rapamycin treatment inhibited insulin-induced PKM2 and HIF-1α expression, and glucose consumption and lactate production. (A)HepG2 and HL7702 cells were transfected with miR-99a and miR-SCR, and treated as in Fig. 2. The expression levels of HIF-1α, PKM2 and β-actin were analyzed by immunoblotting. (B) Relative densities were determined as above and normalized to β-actin expression level. Data were presented by mean±SE (n = 3). The data were analyzed by ANOVA. * and **, indicate significant decrease compared to the control (p<0.05 and p<0.01); ^#^ and ^##^, indicate significant decrease compared to miR-SCR and insulin treatment (p<0.05 and p<0.01). (C) Cells were pretreated with rapamycin (5 nM) for 30 min followed by insulin treatment for 6 h. The protein levels of mTOR, HIF-1α, PKM2and β-actin were detected by immunoblotting. (D) Relative densities of protein expression above. The data were analyzed by ANOVA, ** indicates significant decrease compared to the control (p<0.01); ^#^ and ^##^, indicate significant decrease compared to DMSO and insulin treatment (p<0.05 and p<0.01). (E) Glucose assay and (F) Lactate assay of cells treated as in Fig. 2C. *, indicates significant decrease compared to the control (p<0.05); ^#^ and ^##^, indicate significant decrease compared to DMSO and insulin treatment (p<0.05 and p<0.01).

Recent studies have demonstrated that PKM2 can be regulated by mTOR pathway [30], [31]. In order to study whether mTOR is required for PKM2 in regulating insulin-inducing glucose consumption, we found that inhibition of mTOR by miR-99a overexpression significantly suppressed the downstream molecules HIF-1α and PKM2 expression under the insulin treatment (Figs. 3A and 3B). Similarly, rapamycin, the mTOR inhibitor, also strongly attenuated insulin-induced HIF-1α and PKM2 expression in both HepG2 and HL7702 cells (Figs. 3C and 3D). Rapamycin also decreased insulin-induced glucose consumption and lactate production in these cells (Figs. 3E and 3F). These results demonstrate that insulin induces glucose consumption and lactate production through mTOR/PKM2 pathway, which can be blocked by miR-99a or mTOR inhibitor.

### Knockdown of HIF-1α Inhibited PKM2 Expression and Insulin-induced Glucose Consumption

**Figure 4:**
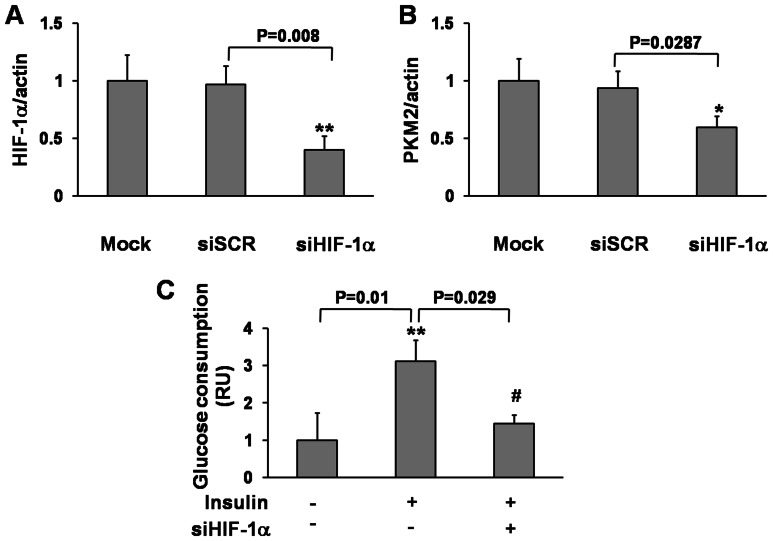
siHIF-1α inhibited HIF-1α and PKM2 expression and insulin-induced glucose consumption. HepG2 cells were transfected with siHIF-1α (40 nM) and siSCR for 24 h. Protein levels of HIF-1α (A) and PKM2 (B) were analyzed by immunoblotting. Histogram represents the levels of HIF-1α and PKM2 normalized to β-actin (mean±SEM; n = 3). * and **, indicate significant decrease compared to the control (p<0.05 and p<0.01). (C) HepG2 cells were transfected with siHIF-1α and siSCR for 24 h, then starved for 18 h followed by insulin treatment for 6 h. Glucose assay of cells was tested as above. The data were analyzed by ANOVA. **, indicates significant increase compared to the control without insulin treatment (p<0.01); ^#^, indicates significant decrease compared to siSCR and insulin treatment (p<0.05).

PKM2 regulates glucose metabolism by functioning as a PHD3-stimulated coactivator for hypoxia-inducible factor 1 in cancer cells [29], [42]. A recent study has demonstrated that mTOR up-regulated of PKM2 expression via hypoxia-inducible factor 1α (HIF-1α)-mediated transcription activation [30]. To determine whether HIF-1α is required for insulin-regulated glucose consumption through PKM2, we knockdown HIF-1α by siRNA approach. Transfection with siHIF-1α in HepG2 decreased HIF-1α protein expression to 40% and reduced PKM2 expression by 40% when compared to the scrambled control (Figs. 4A–C). In addition, HIF-1α Knockdown almost abolished insulin-induced glucose consumption in HepG2 cells (Fig. 4C), suggesting that HIF-1α is necessary for PKM2 in insulin-induced glucose consumption.

## Discussion

mTOR pathway plays a central role in regulating protein synthesis, ribosomal protein translation, and cap-dependent translation [16], [43], [44], [45], [46], [47]. mTOR is recently demonstrated to regulate aerobic glycolysis in cancer cells and promote tumor growth through up-regulation of PKM2 [30], [31]. PKM2 is a ubiquitous prototype enzyme present in embryonic tissues and adult dividing/tumor cells [48]. PKM2 play a critical role in tumor development through its two functions: acting as glycolytic enzyme and protein-kinase-phosphorylating histone [25]. In addition to its primary function to catabolize glucose, PKM2 is the last and the main rate-limiting enzyme in glycolytic pathway [26]. PKM2 is a multifunctional protein and is associated with metabolic regulation, cellular growth, apoptosis and immunological responses [48], [49]. Our study demonstrated that insulin significantly inhibited miR-99a expression and induced mTOR expression, while forced expression of miR-99a was sufficient to inhibit insulin-induced mTOR expression. In addition, miR-99a enhanced the efficacy of photofrin based photodynamic in human glioblastoma [50]. In esophageal squamous cell carcinoma, miR-99a was significantly decreased, and forced expression of miR-99a inhibited cell proliferation by inducing apoptosis [51]. Up to now, it is unclear about the role of miR-99a in insulin-regulated glycolysis.

This study provides a link between insulin and mTOR protein expression regulated by miRNA. In this study, we also showed that insulin induced PKM2 expression and glucose consumption and lactate production, which was regulated by miR-99a/mTOR. This result suggests that PKM2 is a downstream molecule of miR-99a via targeting mTOR, and is involved in insulin-induced glucose metabolism.

Hypoxia-induced factor 1 (HIF-1) is a transcriptional factor consisting of HIF-1α and HIF-1β subunits. HIF-1α mediates essential homeostatic responses to cellular and systemic hypoxia by activating transcription of multiple genes including those encoding erythropoietin, glycolytic enzymes and vascular endothelial growth factor (VEGF) [52], [53]. HIF-1 is known to be involved in insulin signaling. For example, insulin-like growth factor-1 (IGF-1) promotes cell survival, proliferation and angiogenesis via inducing HIF-1α expression [54], [55]. Our previous studies have demonstrated HIF-1α is regulated via PI3K/AKT signal pathway in cancer cells [39], [56]. In this study, we showed that HIF-1α is repressed by miR-99a overexpression through inhibiting mTOR and it is involved in glucose consumption in response to insulin treatment. PKM2 are found to regulate glucose metabolism by coactivating with HIF-1α [29], [42]. A recent study has demonstrated that mTOR up-regulation of PKM2 expression is through HIF-1α-mediated transcription activation and c-Myc-heterogeneous nuclear ribonucleoproteins-dependent regulation of PKM2 gene splicing [30]. In the present study, we found that miR-99a/mTOR/PKM2 pathway is involved in insulin-induced glucose metabolism, and mTOR and HIF-1α are necessary for insulin-induced PKM2 expression and glucose consumption. The results, for the first time, reveal the important role of miR-99a in glycolysis under insulin treatment.

**Figure 5:**
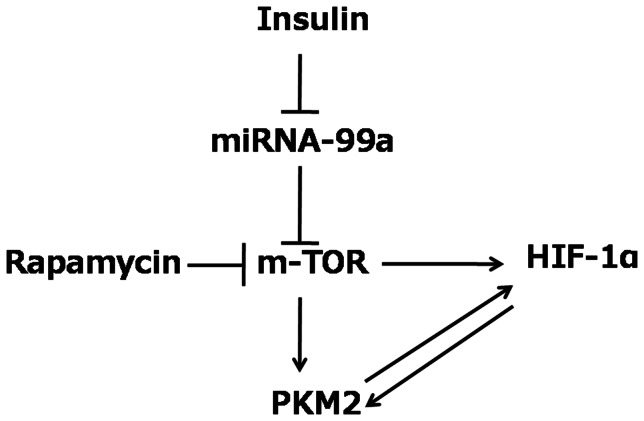
Outline of pathway in insulin-induced glucose consumption. Insulin inhibits the expression of miR-99a, then induces miR-99a direct target mTOR which in turn increases PKM2 and HIF-1α expression for regulating glucose consumption. mTOR inhibitor Rapamycin can inhibit this pathway.

In summary, our study demonstrates that insulin regulates cell glycolysis via inhibition of miR-99a expression in liver cells, and miR-99a/mTOR/HIF-1 is important in insulin-regulated PKM2 expression and glucose consumption (Fig. 5). This finding provides a novel understanding to the molecular mechanism for biological function of insulin in cells.

## Supporting Information

Figure S1 **Insulin induced phosphorylated mTOR (p-mTOR) expression levels, while** **overexpression of miR-99a and rapamycin treatment inhibited insulin-induced p-mTOR levels.** (A) HepG2 and HL7702 cells were starved in serum-free medium for 18 h, then treated with insulin (200 nM) for 6 h. Cells were collected and subjected to immunoblotting using antibodies against p-mTOR and GAPDH. (B) The cells were transfected with miR-99a (+) or miR-SCR (–) precursor, and cultured for 60 hours, then treated with or without insulin as above. The expression levels of p-mTOR and GAPDH were analyzed by immunoblotting. (C) Cells were pretreated with rapamycin (5 nM) for 30 min, followed by insulin treatment for 6 h. The protein levels of p-mTOR and GAPDH were detected by immunoblotting.
(PPTX)
