# Supplementary material for: Insulin Promotes Glucose Consumption via Regulation of miR-99a/mTOR/PKM2 Pathway
Source: PLoS One. 2013 Jun 10;8(6):e64924. doi: 10.1371/journal.pone.0064924 (PMC3677911; doi:10.1371/journal.pone.0064924)
Supplement: Figure S1 — Insulin induced phosphorylated mTOR (p-mTOR) expression levels, while overexpression of miR-99a and rapamycin treatment inhibited insulin-induced p-mTOR levels. (A) HepG2 and HL7702 cells were starved in serum-free medium for 18 h, then treated with insulin (200 nM) for 6 h. Cells were collected and subjected to immunoblotting using antibodies against p-mTOR and GAPDH. (B) The cells were transfected with miR-99a (+) or miR-SCR (–) precursor, and cultured for 60 hours, then treated with or without insulin as above. The expression levels of p-mTOR and GAPDH were analyzed by immunoblotting. (C) Cells were pretreated with rapamycin (5 nM) for 30 min, followed by insulin treatment for 6 h. The protein levels of p-mTOR and GAPDH were detected by immunoblotting. (PPTX) [file pone.0064924.s001.pptx]

## Slide 1
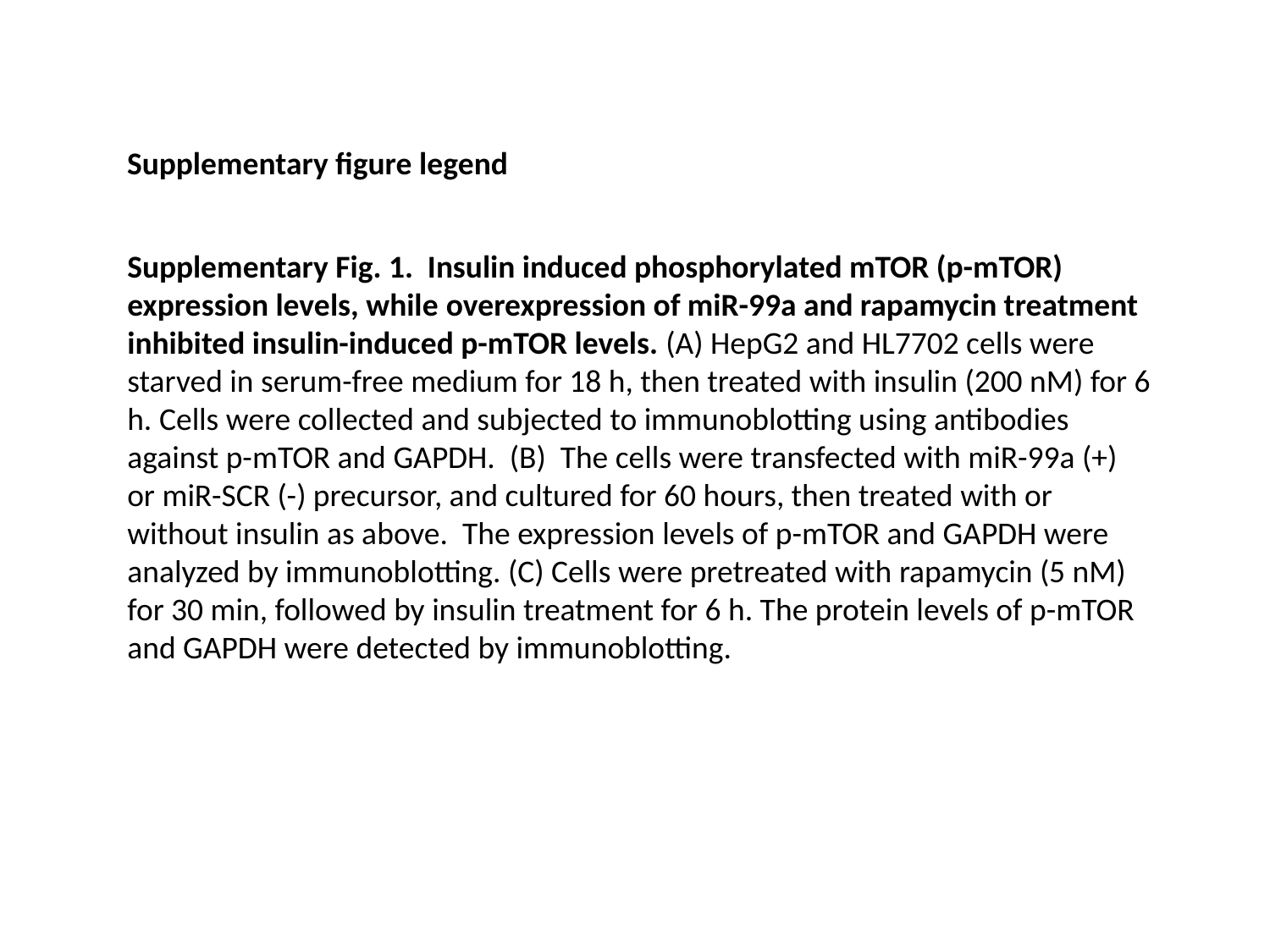

Supplementary figure legend
Supplementary Fig. 1. Insulin induced phosphorylated mTOR (p-mTOR) expression levels, while overexpression of miR-99a and rapamycin treatment inhibited insulin-induced p-mTOR levels. (A) HepG2 and HL7702 cells were starved in serum-free medium for 18 h, then treated with insulin (200 nM) for 6 h. Cells were collected and subjected to immunoblotting using antibodies against p-mTOR and GAPDH. (B) The cells were transfected with miR-99a (+) or miR-SCR (-) precursor, and cultured for 60 hours, then treated with or without insulin as above. The expression levels of p-mTOR and GAPDH were analyzed by immunoblotting. (C) Cells were pretreated with rapamycin (5 nM) for 30 min, followed by insulin treatment for 6 h. The protein levels of p-mTOR and GAPDH were detected by immunoblotting.

## Slide 2
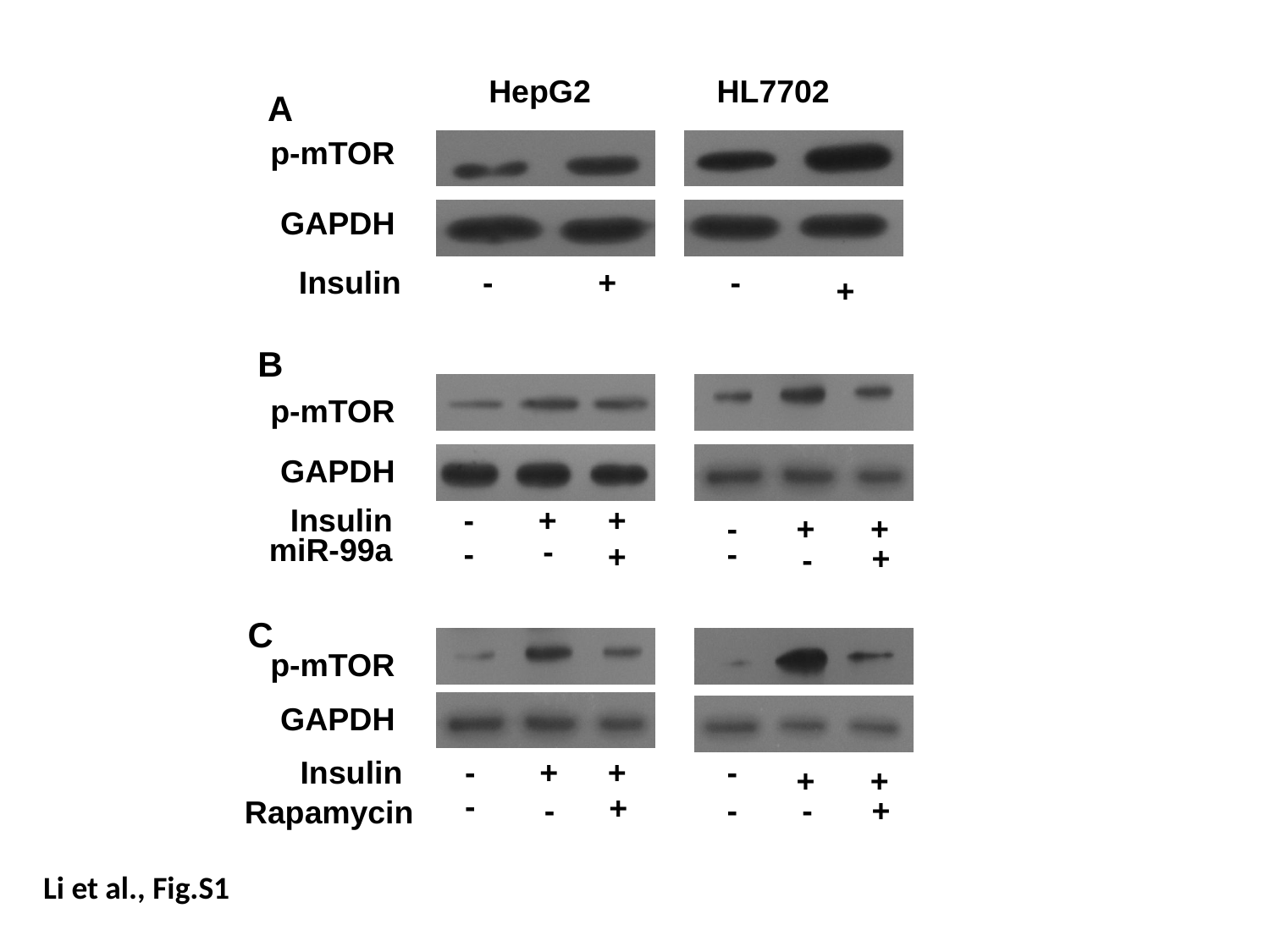

HepG2
HL7702
A
p-mTOR
GAPDH
Insulin
-
+
-
+
B
p-mTOR
GAPDH
Insulin
-
+
+
-
+
+
miR-99a
-
-
-
+
+
-
C
p-mTOR
GAPDH
Insulin
-
+
+
-
+
+
-
+
-
-
-
+
Rapamycin
Li et al., Fig.S1
